# Rac1 conditional deletion attenuates retinal ganglion cell apoptosis by accelerating autophagic flux in a mouse model of chronic ocular hypertension

**DOI:** 10.1038/s41419-020-02951-7

**Published:** 2020-09-10

**Authors:** Meng-Lu Zhang, Guo-Li Zhao, Yu Hou, Shu-Min Zhong, Lin-Jie Xu, Fang Li, Wei-Ran Niu, Fei Yuan, Xiong-Li Yang, Zhongfeng Wang, Yanying Miao

**Affiliations:** Department of Ophthalmology, State Key Laboratory of Medical Neurobiology and MOE Frontiers Center for Brain Science, Institutes of Brain Science, Zhongshan Hospital, Fudan University, 200032 Shanghai, China

**Keywords:** Autophagy, Retina

## Abstract

Autophagy has a fundamental role in maintaining cell homeostasis. Although autophagy has been implicated in glaucomatous pathology, how it regulates retinal ganglion cell (RGC) injury is largely unknown. In the present work, we found that biphasic autophagy in RGCs occurred in a mouse model of chronic ocular hypertension (COH), accompanied by activation of Rac1, a member of the Rho family. Rac1 conditional knockout (Rac1 cKO) in RGCs attenuated RGC apoptosis, in addition to blocking the increase in the number of autophagosomes and the expression of autophagy-related proteins (Beclin1, LC3-II/I, and p62) in COH retinas. Electron micrograph and double immunostaining of LAMP1 and LC3B showed that Rac1 cKO accelerated autolysosome fusion in RGC axons of COH mice. Inhibiting the first autophagic peak with 3-methyladenine or Atg13 siRNA reduced RGC apoptosis, whereas inhibiting the second autophagic peak with 3-MA or blocking autophagic flux by chloroquine increased RGC apoptosis. Furthermore, Rac1 cKO reduced the number of autophagosomes and apoptotic RGCs induced by rapamycin injected intravitreally, which suggests that Rac1 negatively regulates mTOR activity. Moreover, Rac1 deletion decreased Bak expression and did not interfere with the interaction of Beclin1 and Bcl-2 or Bak in COH retinas. In conclusion, autophagy promotes RGC apoptosis in the early stages of glaucoma and results in autophagic cell death in later stages. Rac1 deletion alleviates RGC damage by regulating the cross talk between autophagy and apoptosis through mTOR/Beclin1-Bak. Interfering with the Rac1/mTOR signaling pathway may provide a new strategy for treating glaucoma.

## Introduction

Autophagy, a basic life phenomenon of eukaryotic cells, has a physiological role by removing damaged organelles in cells and promoting cell survival^1^. There is also evidence implicating autophagy in neurodegenerative diseases^2^, including glaucoma^3–5^.

Glaucoma, a progressive and irreversible optic neuropathy characterized by loss of the visual field, is due to retinal ganglion cell (RGC) apoptotic death. High intraocular pressure (IOP) is considered as the most important risk factor for glaucoma^6^. Although numerous autophagosomes have been found in retinas, and the autophagy-related genes LC3 and Beclin1 are upregulated in experimental models of glaucoma^4,5,7^, the role of autophagy in glaucomatous RGC injury is still controversial. In a rat model of chronic ocular hypertension (COH), inhibition of autophagy reduced RGC apoptosis^8^. By contrast, autophagy induced by the mTOR inhibitor rapamycin protected RGCs from death in COH rats^9^ and slowed down the degradation of optic nerve axons^10^. Therefore, it is necessary to clarify the precise relationship of autophagy and RGC apoptotic death in glaucoma.

Autophagy is a multi-step process from nucleation, elongation, autophagosome to autolysosome formation, which is precisely regulated by a series of molecules. Actin dynamics have important roles throughout the various steps of autophagy^11^. Given that the pivotal role of the Rho family of small G proteins in the regulation of cytoskeletal dynamics, Rac1, a member of the Rho family was intensively studied its function in autophagy. mTOR, a serine/threonine-protein kinase, is an initiating molecule by the formation of ULK complex composed of ULK, Atg13, and FIP200 when its activity is inhibited^1,12,13^. Rac1 binds mTOR directly to regulate mTOR activity and subcellular localization^14,15^, thereby regulating autophagy^15–17^. Rac1 is also widely expressed in mice retinas^18,19^. Retinal quantitative proteomics in glaucomatous animal models showed increased Rac1 expression^20^. Therefore, we hypothesize that Rac1 maybe have a role in RGC injury by regulating autophagy in COH retinas. In this study, we show that Rac1deleltion mitigates RGC apoptosis by accelerating autophagic flux, which is found to be dual phases and has different roles at different stages in COH retinas. The mTOR-Beclin1/Bak signaling pathway is involved in Rac1-mediated autophagy.

## Materials and methods

### Animals

All animals in this experiment were treated according to the National Institutes of Health (NIH) guidelines for the Care and Use of Laboratory Animals and the guidelines of Fudan University on the ethical use of animals to minimize the pain caused by experiments. Adult male C57BL/6J mice (7–8 weeks old, 18–22 g) were purchased from SLAC Laboratory Animal Co. Ltd (Shanghai, China). We obtained RGC Rac1 conditional knockout (Rac1 cKO) mice by mating Rac1^fl/fl^ mice (The Jackson Laboratory, Bar Harbor, ME, USA) and Tg(Chat-cre)GM24Gsat mice (gift from Dr. Min-Min Luo at the National Institute of Biological Sciences, Beijing, China), in which Cre recombinase is ectopically expressed in RGCs and dendrites. The Rac1 cKO mouse was described in detail previously^21^.

### COH mouse model

The COH mouse model was established following our previous procedure^22^ with minor modification. Briefly, the mice were anesthetized by 10% chloral hydrate (4 mg/kg, i.p.) and the dose of micro-magnetic beads (diameter ≈ 9 μm BioMag^®^ Superparamagnetic Iron Oxide, Bangs Laboratories, Ins) was 2 μL to inject into the anterior chamber of the right eye only once. The sham group was subjected to the same procedure without the injection of beads.

The measurement of intraocular pressure (IOP) of both eyes was previously described in detail^22^ and performed immediately after surgery (G0d); at day 4 (G4d) and weeks 1, 2, 3, and 4 (G1w, G2w, G3w, and G4w, respectively) after surgery. If the IOP did not increase at G4d, the animals will be excluded from the experiments. The successful COH mice were randomly allocated to different experimental groups.

### Western blotting analyses

Western blotting analyses were performed as described previously^23,24^. The antibodies were listed in Supplementary Table S1.

### Co-immunoprecipitation

We assayed interactions of Beclin1 and Bcl-2 or Bak using a co-IP kit (Pierce^TM^, ThermoScientific, Pittsburgh, PA, USA) according to the manufacturer’s instructions described previously^25,26^. The Bcl-2, Bak, and human IgG (as a negative control) antibodies were purified with a Pierce Antibody Clean-up Kit (ThermoScientific). Pre-cleared lysates were obtained from the retinas of wild-type (wt) or Rac1 cKO mice. The immunoprecipitated complexes were detected by the Beclin1 antibody following western blotting.

### TUNEL detection

RGC apoptosis was detected in whole-mounted retinas with the DeadEnd Fluorometric TUNEL System G3250 kit (Promega, Madison, WI, USA) according to the manufacturer’s instructions^27^. The GCL upward mounted-retina was consecutively scanned for five steps (1 μm/step) to make sure that only GCL was captured. All TUNEL-positive signals merged well with DAPI-labeled nuclei were counted in each retina by two trained independent experimenters who were blind to the animal groups. Images were acquired by an FV1000 confocal laser-scanning microscope and processed with FV10-ASW Viewer 1.7 software (Olympus, Tokyo, Japan) and Adobe Photoshop CC (Adobe Systems, Inc., San Jose, CA, USA).

### Double immunofluorescent staining

Double immunofluorescent staining was conducted as previously described^25^. The antibodies were listed in Supplementary Table S1. Images were digitally acquired by an FV1000 confocal laser-scanning microscope (Olympus, Japan). The retinas of these mice ranged in diameter from 4.4 to 4.8 mm. Two fields, one from the central region (<1.0 mm distant from optic nerve head) and the other from the peripheral region (between 1.0 and 2.0 mm distant from the optic nerve head), were randomly selected at 0, 90, 180, and 270° angles of each retina, respectively (Fig. 2e). Therefore, eight fields were chosen from one retina and the number of LAMP1 and LC3B double-positive cells was counted by two independent experimenters who were blind to the animal groups.

### Autophagosome detection

The autophagosomes in RGCs were detected with an autophagy detection kit according to the manufacturer’s instructions (ab139484, Abcam). Whole retinas were completely and quickly removed into artificial cerebrospinal fluid bubbled with oxygen (95% O_2_, 5% CO_2_) for 30 min and transferred to mixed dyes of green detection reagent (1:1000) and Hoechst 33342 (1:1000) for autophagosome and nuclei staining, respectively, for 30 min at room temperature. After being washed with assay buffer four times, the whole retinas were unfolded on a glass slide and covered with a coverslip. The number of autophagosomes in the whole retinas was counted under the FV1000 confocal laser-scanning microscope by two independent experimenters who were blind to the animal groups. Images were processed with FV10-ASW Viewer 1.7 and Adobe Photoshop CC.

### Intravitreal injection

Intravitreal injection was performed as described previously^22,28^. We injected rapamycin of 1 μL (2 μg/μL, Merck, Darmstadt, Germany) or Atg13 siRNA with sequences of sense 5′-CUCACUCUUUCCUGUGCUUdTdT-3′ and antisense 5′-AAGCACAGGAAAGAGUGAGdTdT-3′ of 1 μL (5 nM diluted in 10 μL normal saline, RiboBio Co., LTD, Guangzhou, China) or negative control (NC) siRNA in the same volume slowly to avoid fundus blood vessels using a Hamilton syringe under an ophthalmic surgical microscope.

### Pharmacological treatment

Mice were injected intraperitoneally with 3-methyladenine (3-MA, 2.5 mM in 0.35 ml, 26.1 mg/kg, Sigma-Aldrich) at G3d or G18d, 600 μM chloroquine diphosphate (CQ, 11.65 mL/kg, 2.24 mg/kg, Abcam) at G18d; Bafilomycin A1 (Baf-A1, 0.1 mg/kg, i.p. Cell Signaling Technology) 1 day before surgery for assay at G4d, and additional dose (0.1 mg/kg, i.p.) once at G2w for assay at G3w. Control mice were injected with the same volume of normal saline (NS) in the same way.

### Transmission electron microscopy

Optic nerves ~3 mm from the eyeballs were dissected from mice retinas and fixed in 2.5% glutaraldehyde fixative at 4 °C overnight, then post-fixed with 1% osmium tetroxide for 1.5 h at room temperature. After standard ethanol dehydration, samples were embedded in an Epon-Araldite 812 mixture. Ultrathin (70-nm-thick) and semi-thin sections (700-nm-thick) were obtained with a Reichert Ultracut S ultratome (Leica, Nussolch, Germany) for transmission electron microscopy and toluidine blue staining, respectively. After staining with uranyl acetate and lead citrate, samples were observed with a CM120 transmission electron microscope (Philips, Netherlands). Autophagosome was identified by double-membrane with electron-dense material, and autolysosome was characterized with one or two limiting membranes with an uneven mass of electron-dense materials and/or vacuoles in axons, which were counted in at least five random fields (7.5 µm × 4.8 µm) per sample by two independent and experienced investigators who were blind to the animal groups.

### Statistical analyses

All data in this experiment are expressed means ± standard errors (means ± S.E.M). Statistical data were analyzed with GraphPad Prism (version 6.0; GraphPad Software, San Diego, CA, USA). Student’s *t*-test and one-way ANOVA with Tukey’s multiple comparison test was used to compare data from two or multiple groups, respectively. *P*-values < 0.05 were considered statistically significant.

## Results

### Biphasic autophagy occurred in COH retinas accompanied by Rac1 activation

We successfully established the COH mouse model. The IOP in the sham group (control, Ctr) was 13.4 ± 0.1 mmHg (*n* = 66). There was no difference in IOP between the left and right eyes of the mice before G0d. The IOP of the right eyes increased to 23.4 ± 0.3 mmHg (*n* = 132, *P* < 0.001 vs. Ctr) at G4d and remained at high levels from G1w to G4w.

**Fig. 1:**
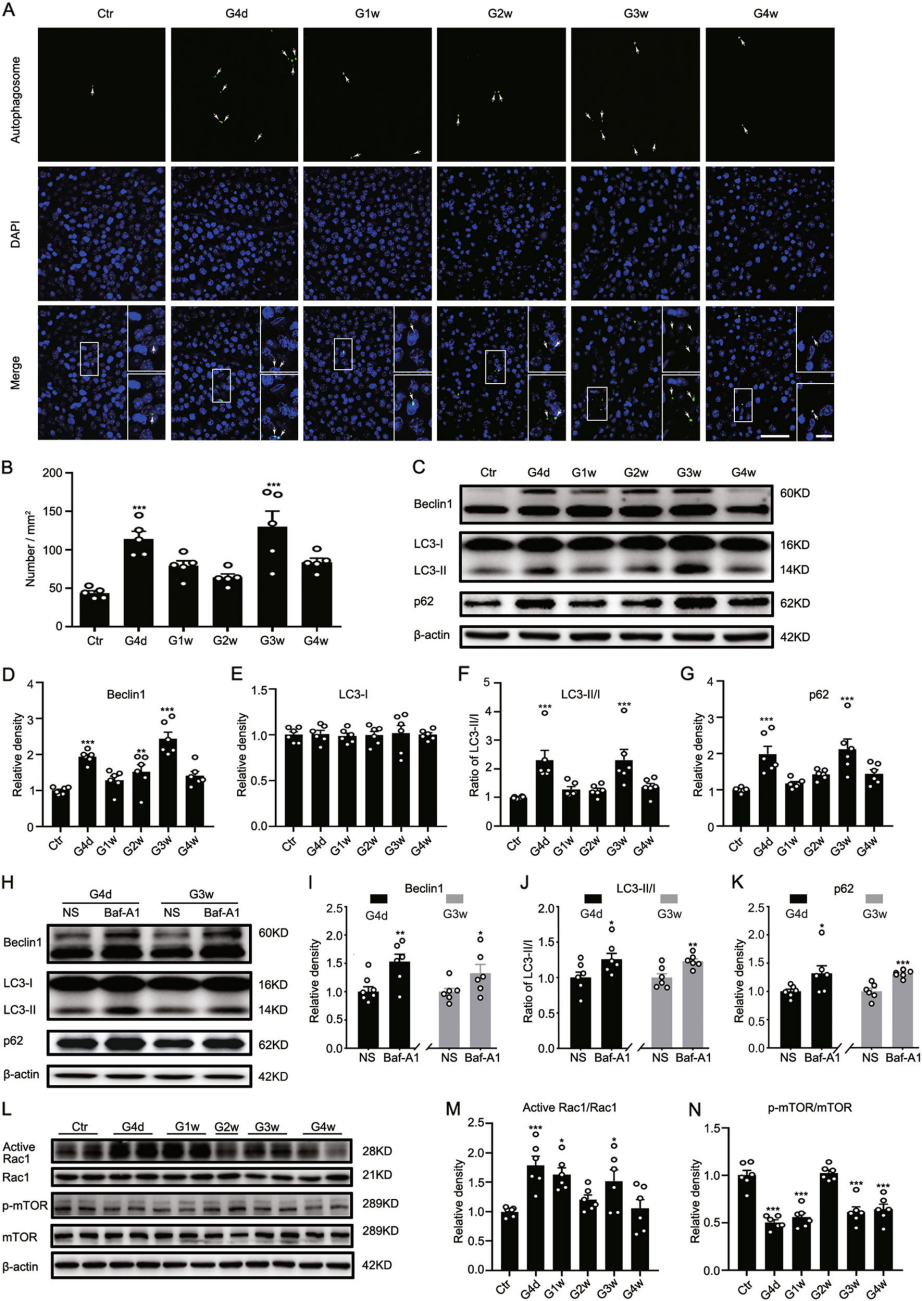
Biphasic autophagy and Rac1 activation in COH retinas. **a** Representative confocal images showing the changes in the number of autophagosomes in sham-operated (Ctr) and COH whole-mounted retinas at different post-operational time (G4d-G4w). The insets on the right sides of merged images are the enlarged images of the white squares. Arrows indicate the positive signals of autophagosomes. Scale bar: 50 µm for all images except the enlarged images (15 µm). **b** Bar chart summarizing the average numbers of autophagosomes per mm^2^ in whole-mounted retinas under different conditions. Error bars represent S.E.M., and *n* = 5 for each group. **c** Representative immunoblots showing the changes in Beclin1, LC3-II/I, and p62 expression in control (Ctr) and COH retina extracts at different post-operational time (G4d-G4w). **d**–**g** Bar charts summarizing the average densitometric quantification of immunoreactive bands of **d** Beclin1, **e** LC3-I, **f** LC3-II/I, and **g** p62 in Ctr and COH retinas, respectively. **h** Representative immunoblots showing the changes in Beclin1, LC3-II/I, and p62 expression in NS (as control)- or bafilomycin A1- (Baf-A1) treated retinal extracts at G4d and G3w. **i**–**k** Bar charts summarizing the average densitometric quantification of immunoreactive bands of **i** Beclin1, **j** LC3-II/I, and **k** p62, respectively. **l** Representative immunoblots showing the changes in active Rac1, Rac1, mTOR, and p-mTOR expression in Ctr and COH retina extracts at different post-operational time (G4d-G4w). **m**, **n** Bar charts summarizing **m** the average active Rac1/Rac1 and **n** p-mTOR/mTOR ratios under the condition of **l**, respectively. All data are normalized to their corresponding β-actin data and then to Ctr. Error bars represent S.E.M., and *n* = 6 for all the groups. *P < 0.05, **P < 0.01, ***P < 0.001 vs. Ctr or NS.

We first detected autophagosomes in COH retinas. Few autophagosomes were observed in control retinas (Ctr). Numerous autophagosome-positive signals were observed in COH retinas, with two peaks at G4d and G3w (Fig. 1a, b). The nuclei of autophagic neurons were reduced in size and more concentrated at G3w and G4w (shown in enlarged images) (Fig. 1a).

Consistent with the autophagosome change, autophagy-related proteins Beclin1 and p62, as well as the LC3-II/I ratio increased significantly at G4d and G3w (*n* = 6, all *P* < 0.001 vs. Ctr) (Fig. 1c, d, f, g). The protein levels of LC3-I in COH retinas were similar to that of the control group (Fig. 1e).

In order to exclude the possibility that the increase of LC3-II, p62, and Beclin1 is due to the blockage of autophagic degradation, we treated the COH mice with Baf-A1, an inhibitor for blocking the fusion of autophagosomes and lysosomes, and found that the protein levels of Beclin1, LC3-II/I and p62 were further increased after Baf-A1 treatment (Fig. 1h–k). The above results demonstrated that RGC autophagy was activated with two peaks by elevated IOP.

Next, we found that changes in the ratio of active Rac1 to Rac1 paralleled with those of autophagy in COH retinas (Fig. 1l, m). That is, two peaks appeared at G4d and G3w, which suggests that Rac1 may be implicated in the regulation of autophagy in glaucoma. Our results showed that the ratio of phosphorylated mTOR (p-mTOR) to mTOR decreased from G4d, then returned to the control level at G2w and decreased again from G3w (Fig. 1l, n). Thus, decreased mTOR activity may result in the initiation of autophagy.

### Rac1 deletion reduces RGC autophagy and apoptosis in COH retinas

**Fig. 2:**
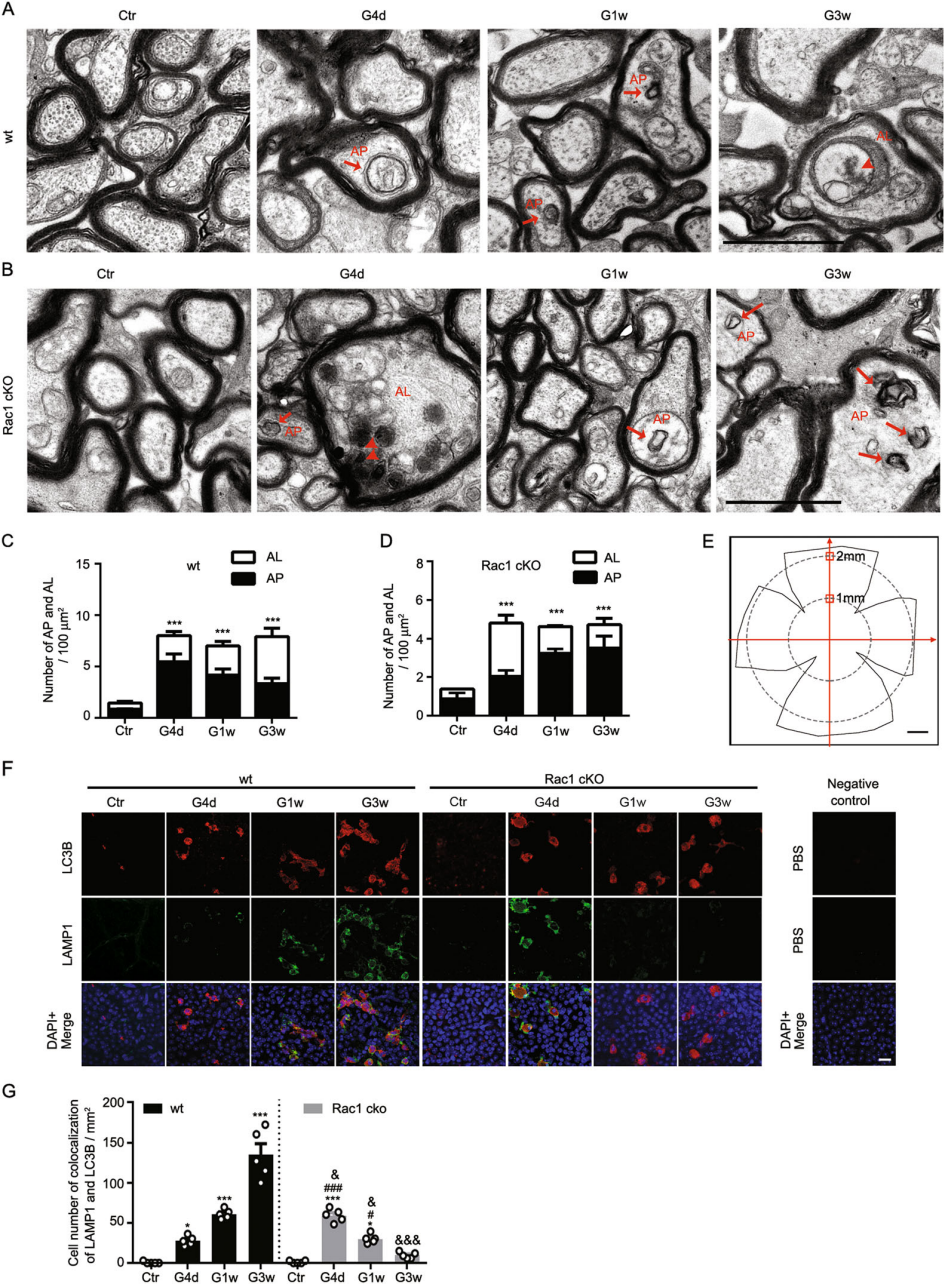
Rac1 deletion in RGC accelerates the fusion of autophagosomes with lysosomes in RGC axons of COH mice. **a**, **b** Representative electronic micrographs showing the expression of autophagosome-like structures (AP) and autolysosome-like structure (AL) in RGC axons in **a** COH wt and **b** COH Rac1 cKO mice at different post-operational time. The arrows indicate AP and the arrowheads indicate the electron-dense in AL. Scale bar: 1 µm for all images. **c**, **d** The bar charts showing the average numbers of AP and AL per unit area of electronic micrographs as shown in **a** and **b**, respectively. ***P < 0.001 vs corresponding control (Ctr); error bars represent S.E.M. and *n* = 5 for wt group, and *n* = 4 for the Rac1 cKO group. **e** Schematic diagram showing the selected areas for counting the number of LAMP1 and LC3B double-positive cells. Scale bar: 500 μm. **f** Representative images showing the expressions of LAMP1 and LC3B by double immunostaining in whole-mounted retinas of COH wt and COH Rac1 cKO mice at different post-operational time; n = 5 for each group; Scale bar: 20 μm for all images. **g** Bar charts showing the average numbers of LAMP1 and LC3B double-positive cells as shown in **f**. Error bars represent S.E.M. *P < 0.05, ***P < 0.001 vs. wt Ctr; ^#^*P* < 0.05, ^###^*P* < 0.001 vs. Rac1 cKO Ctr; ^&^*P* < 0.05, ^&&&^*P* < 0.001 vs. wt at corresponding time points.

Since the autophagy in COH retinas showed two peaks, we selected the peak time to observe the effect of Rac1 on retinal autophagy in mice with Rac1 cKO in RGCs by electron microscopy. The average IOP in Rac1 cKO mice with or without COH was not different from those of wt mice at the same time points. The expression of retinal Rac1 in our Rac1 cKO mouse has been detected in our previous study^21^. As shown in Fig. 2a, axons of wt mice were arranged closely with uniform thickness and an intact myelin sheath; complete mitochondrial structures were observed in the control sample (Ctr). A few autophagosome-like (AP) structures with bilayer membrane coating were found at G4d, AP structures, and swollen axonal mitochondria were observed at G1w, and a large number of autolysosome-like (AL) structures and vacuoles were observed at G3w in COH mice. However, in Rac1 cKO mice, AL structures were found at G4d after IOP elevation, and AP structures were observed from G1w (Fig. 2b). The total number of AP and AL was significantly increased in axons of both COH wt and Rac1 cKO mice at all time points investigated as compared to their controls (Fig. 2c, d). The percentage of AL in the total number of AP and AL in Rac1 cKO axons at G4d was significantly elevated (*P* < 0.001 vs. wt G4d). Furthermore, the results of double immunostaining of LAMP1, a lysosomal marker, and LC3B revealed an increase in the number of double-positive cell in wt retinas and a decrease in Rac1 cKO retinas from G4d to G3w. At G4d, the number of double-positive cell in Rac1 cKO retinas was significantly higher than that of wt retinas (*n* = 5, *P* = 0.017 vs. wt G4d) (Fig. 2f, g). These results suggest that Rac1 deletion accelerates the fusion of autophagosomes and lysosomes in RGCs of COH mice.

**Fig. 3:**
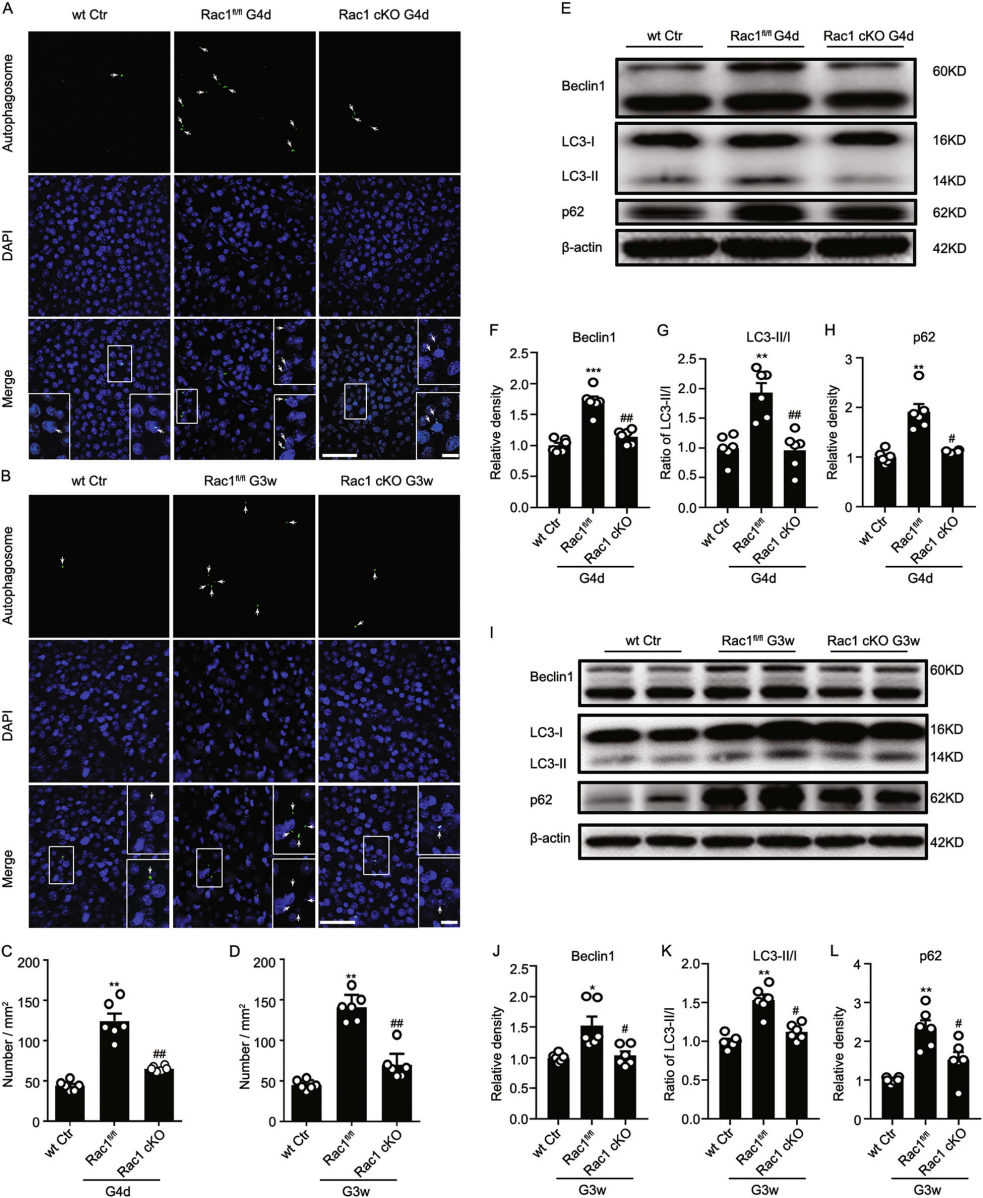
Rac1 deletion in RGCs reduces autophagosomes in COH retinas. **a**, **b** Representative images showing the changes in the number of autophagosomes in wild-type sham (wt, Ctr), Rac1^fl/fl^ and Rac1 cKO whole-mounted retinas with or without COH at **a** G4d or **b** G3w, respectively. The insets at the bottoms of merged images are the enlarged images of the white squares. The arrows indicate the autophagosome-positive signals. Scale bar: 50 µm for all images except the enlarged images (15 µm). **c**, **d** Bar charts showing the average numbers of autophagosomes per mm^2^ in whole-mounted retinas under the conditions as shown in **a** or **b**, respectively. Error bars represent S.E.M., and *n* = 6 for each group. **P < 0.01 vs. wt Ctr; ^##^*P* < 0.01 vs. Rac1^fl/fl^ G4d or G3w. **e** Representative images showing the changes in the expression of Beclin1, LC3-II/I, and p62 in wt, Rac1^fl/fl^ and Rac1 cKO retinas with or without COH for 4 days by western blotting. **f**–**h** Bar charts showing the average densitometric quantification of immunoreactive bands of Beclin1, LC3-II/I, and p62. **i** Representative images showing the changes in the expression of Beclin1, LC3-II/I, and p62 in wt, Rac1^fl/fl^ and Rac1 cKO with or without COH for 3 weeks. **j**–**l** Bar charts showing the average densitometric quantification of immunoreactive bands of Beclin1, LC3-II/I, and p62. All data are normalized to their corresponding β-actin data and then to Ctr. Error bars represent S.E.M., and *n* = 6 for all the groups. *P < 0.05, **P < 0.01, ***P < 0.001 vs. wt Ctr; ^#^*P* < 0.05, ^##^*P* < 0.01 vs. Rac1^fl/fl^ G4d or Rac1^fl/fl^ G3w.

Rac1 cKO also significantly reduced the number of autophagosomes at G4d and G3w (Fig. 3a–d). Rac1 deletion reduced the expressions of Beclin1, LC3-II/I, and p62 in COH retinas at G4d and G3w (Fig. 3e–l). These results suggest that Rac1 deletion decreases autophagosomes in COH retinas by accelerating the degradation of autophagic substances.

### RGC apoptosis peaks between biphasic autophagy in COH retinas

**Fig. 4:**
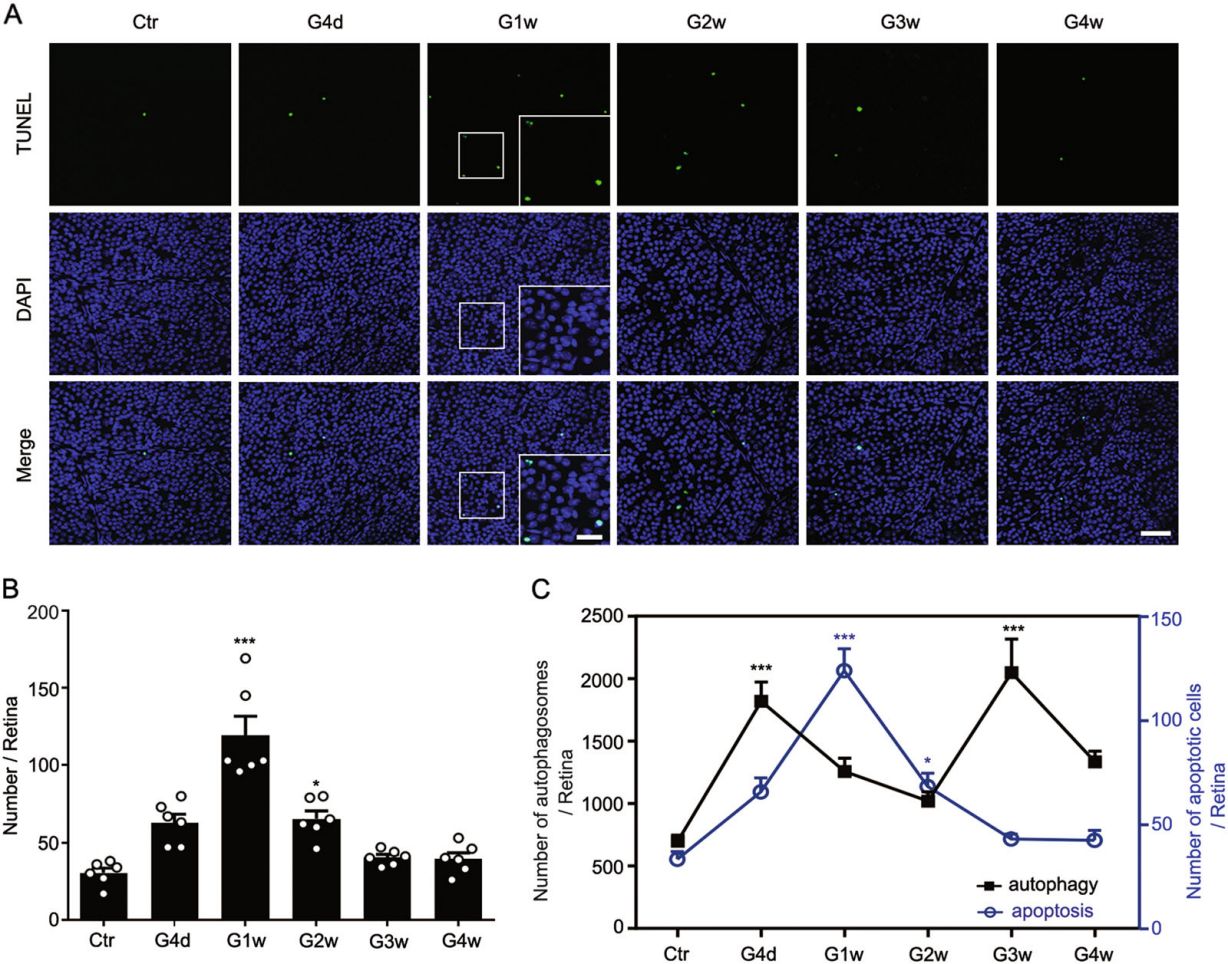
RGC apoptosis in COH retinas. **a** Representative images of TUNEL staining in sham (Ctr) and COH whole-mounted retinas at different post-operational time (G4d-G4w). The insets in the images of the G1w group are the enlarged images of the white squares, respectively. Scale bar: 50 µm for all images except for the enlarged images (20 µm). **b** Bar chart showing the average numbers of TUNEL-positive signals in whole-mounted retinas under different conditions. Error bars represent S.E.M., and *n* = 6 for all the groups. **c** The curve chart showing the relationship between autophagy and apoptosis in RGCs during IOP elevation. *P < 0.05, ***P < 0.001 vs. corresponding Ctr.

Apoptotic signals were scarce in the control group and increased gradually in COH retinas, reaching a peak at G1w (Fig. 4a, b). Figure 4c showed autophagy peaked at G4d and G3w, whereas the apoptosis reached a peak at G1w, which suggests that autophagy precedes RGC apoptosis in COH retinas.

### Different roles of biphasic autophagy in COH retinas

**Fig. 5:**
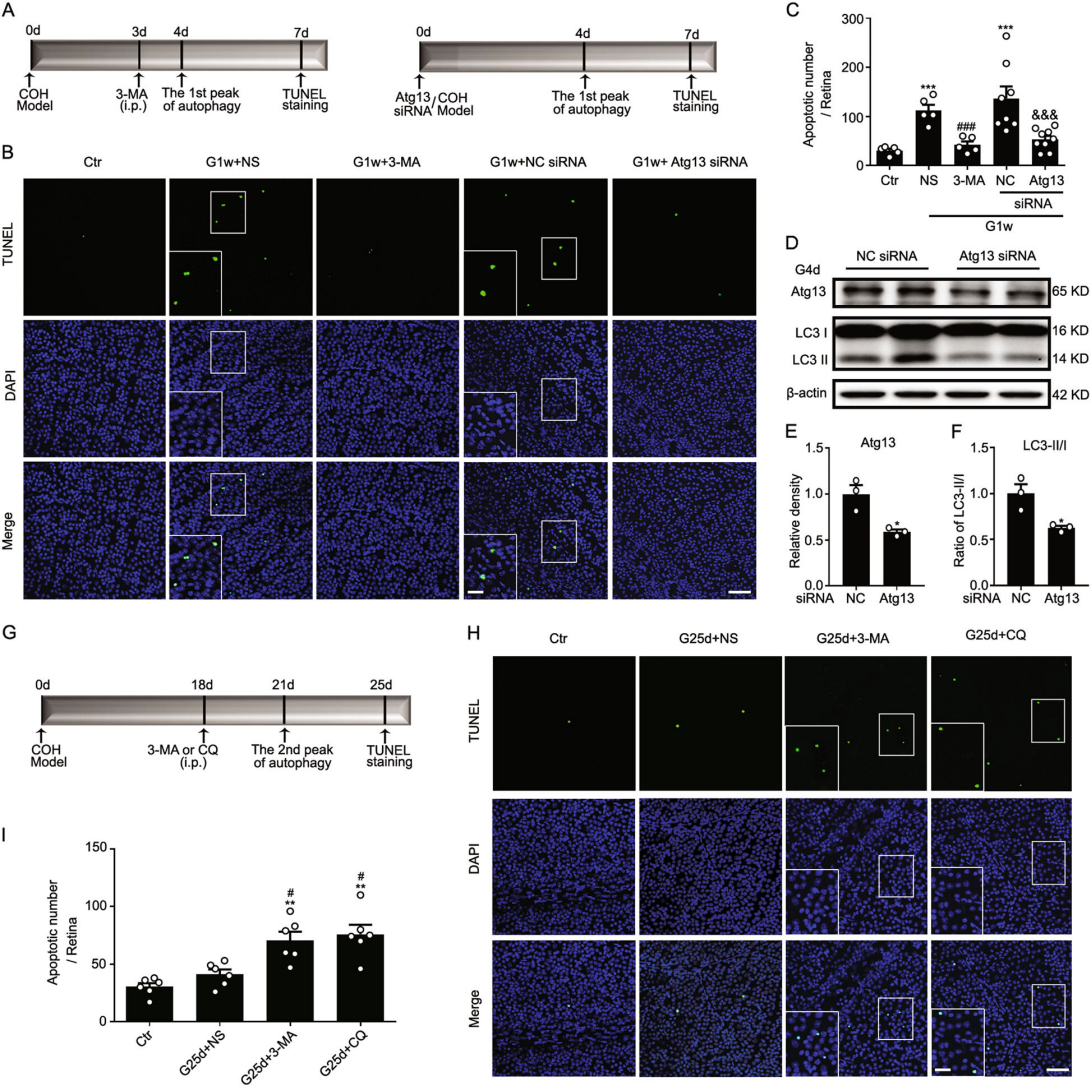
Different roles of biphasic autophagy flux in RGC apoptosis in COH retinas. **a** A schematic diagram showing the procedure for assaying the role of first autophagic flux in RGC apoptosis. **b** Inhibition of autophagy by 3-MA administration at G3d (*n* = 5) or intravitreal injection of Atg13 siRNA (*n* = 9) just before surgery reduced the apoptotic RGC numbers, which was assayed in whole-mounted retinas at G1w by TUNEL staining. The insets in the images of the G1w+NS group are the enlarged images of the white squares, respectively. The scale bar is 50 µm for all images except 20 µm in the enlarged images. **c** Bar chart showing the average number of TUNEL-positive signals in whole-mounted retinas under the conditions as shown in **b**. Error bars represent S.E.M. ***P < 0.001 vs. Ctr, ^###^*P* < 0.001 vs. G1w+NS. ^&&&^*P* < 0.001 vs. G1w+NC siRNA; NS normal saline; NC negative control. **d** Representative immunoblots showing the changes in the expression of Atg13 and LC3-II/I in NC siRNA or Atg13 siRNA treated COH retinal extracts at G4d by western blotting. **e**, **f** Bar charts summarizing the average densitometric quantification of immunoreactive bands of Atg13 and LC3-II/I as shown in **d**, respectively. Error bars represent S.E.M., and *n* = 3. *P < 0.05 vs. G4d+NC siRNA. **g** A schematic diagram showing the procedure for assaying the role of second autophagic flux in RGC apoptosis. **h** Inhibition of autophagy by 3-MA or chloroquine (CQ) administration at G2w increased the apoptotic RGC numbers, assayed in whole-mounted retinas at G25d by TUNEL staining, respectively. The insets in the images of G25d + 3-MA and G25d+CQ groups are the enlarged images of the white squares, respectively. The scale bar is 50 µm for all images except 20 µm in the enlarged images. **i** Bar chart showing the average apoptotic RGC numbers in whole flat-mounted retinas under the conditions as shown in **e**. Error bars represent S.E.M., and *n* = 6 for all the groups. **P < 0.01 vs. Ctr, ^#^*P* < 0.05 vs. G25d+NS.

The possible roles of the two autophagy peaks in COH retinas were explored by pharmacological or genetic methods to block autophagy (Fig. 5a). Our results showed that 3-MA and Atg13 siRNA both significantly reduced the total number of TUNEL-positive RGCs at G1w (Fig. 5b, c). The first peak of autophagy inhibited by Atg13 siRNA injection was testified by the reduction of LC3-II/I (Fig. 5d–f). However, when the second autophagy peak (G3w) was inhibited by 3-MA or CQ at G18d, an increase in RGC apoptosis was detected at G25d (Fig. 5h, i). These results suggest that autophagy in the early stages of COH retinas promotes RGC apoptosis, whereas autophagy in later stages may impede RGC apoptosis.

### Rac1 deletion prevents apoptosis of RGCs in COH retinas

**Fig. 6:**
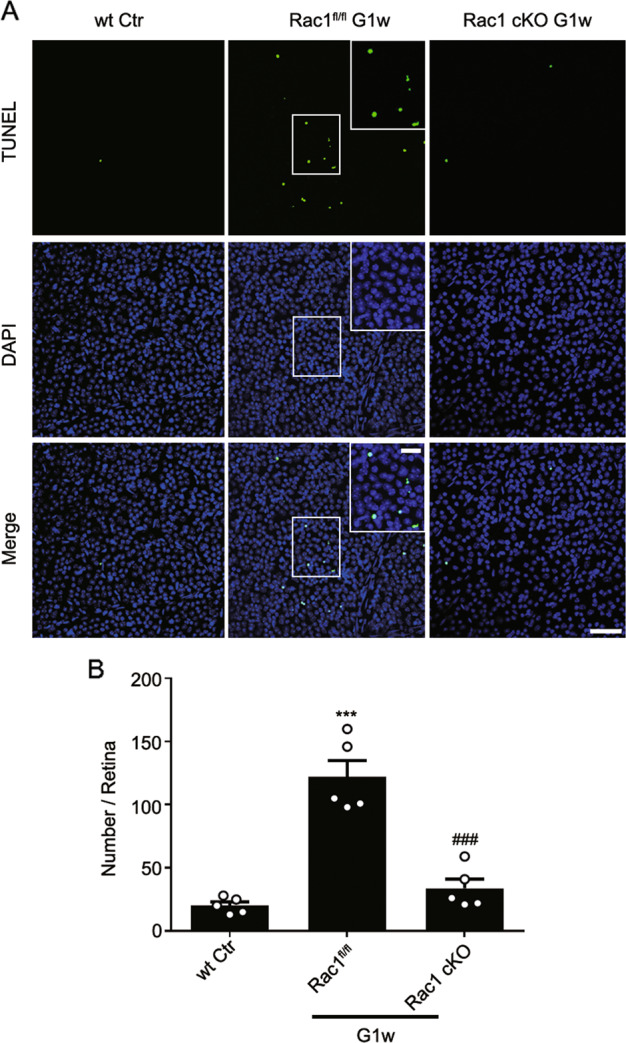
Rac1 deletion in RGCs reduces RGC apoptosis in COH retinas. **a** Representative images of TUNEL staining in wt sham (Ctr), Rac1^fl/fl^ and Rac1 cKO COH retinas, respectively. The insets in the right corners of the images of the Rac1^fl/fl^ G1w group are the enlarged images of the white squares. Scale bar: 50 µm for all images except 20 µm in the enlarged images. **b** Bar chart showing the average numbers of apoptotic RGCs in whole-mounted retinas under different conditions as shown in **a**. Error bars represent S.E.M., and *n* = 5 for all the groups. ***P < 0.001 vs. wt Ctr; ^###^*P* < 0.001 vs. Rac1^fl/fl^ G1w.

The effects of the Rac1 deletion on RGC apoptosis in COH retinas were investigated. Compared to Rac1^fl/fl^ mice at G1w, the number of apoptotic RGCs in Rac1 cKO retinas decreased significantly (Fig. 6), which demonstrates that Rac1 deletion reduces RGC apoptosis in glaucomatous retinas in addition to attenuating autophagy.

### Rac1 deletion blocks rapamycin-induced RGC autophagy and apoptosis

**Fig. 7:**
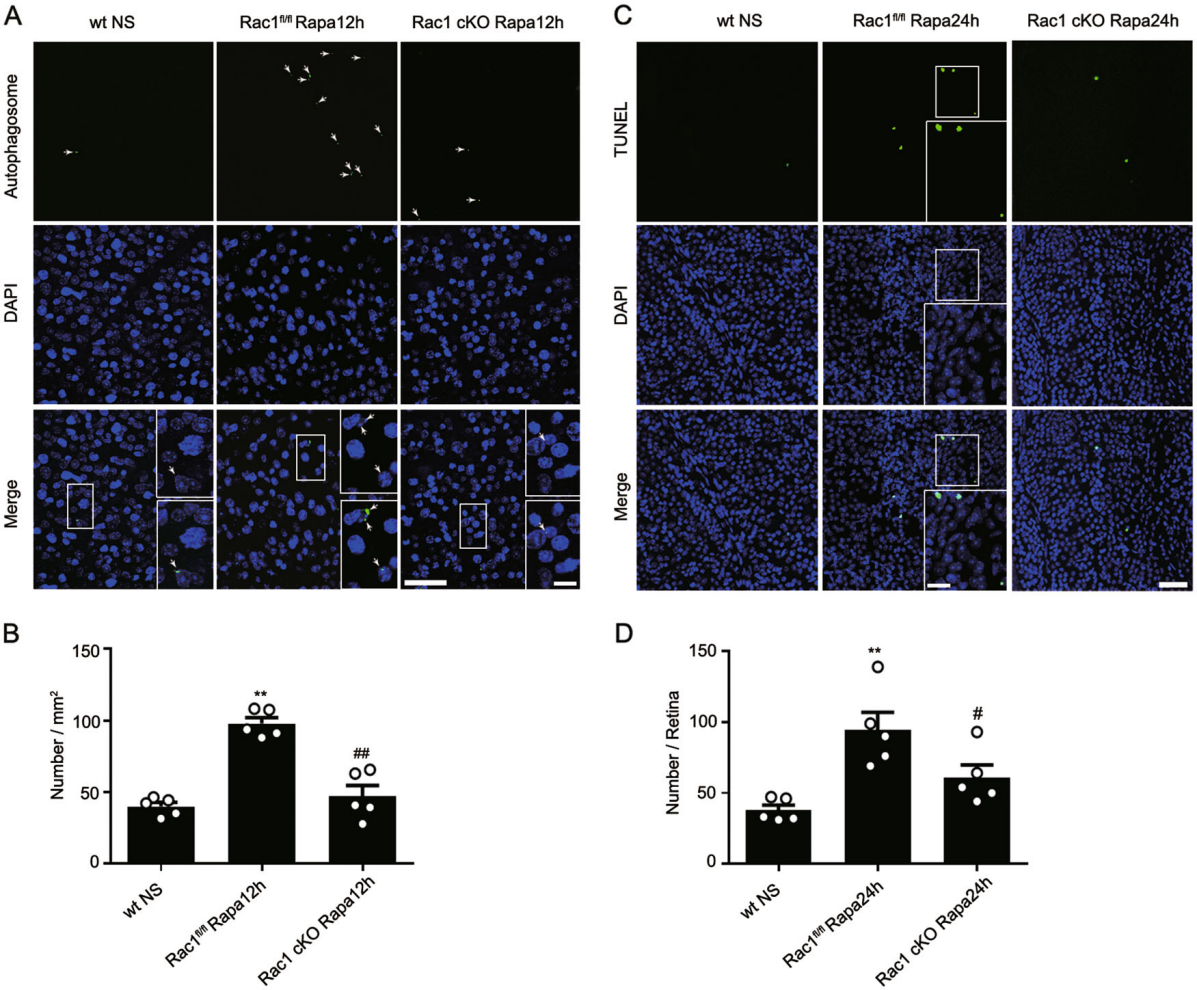
Rac1 deletion in RGCs reduces the rapamycin-induced autophagy. **a** Representative images showing the changes in the number of autophagosome in wt with normal saline treatment (wt NS), Rac1^fl/fl^ or Rac1 cKO retinas 12 h after rapamycin treatment (Rapa12h). The insets in the right of merged images are the enlarged images of the white squares. Scale bar: 50 µm for all images except 10 µm in the enlarged images. **b** Bar chart showing the average numbers of autophagosomes per mm^2^ in whole-mounted retinas under the conditions as shown in **a**. Error bars represent S.E.M., and *n* = 5 for all the groups. **P < 0.01 vs. wt NS; ^##^*P* < 0.01 vs. Rac1^fl/fl^ Rapa12h. **c** Representative images showing RGCs apoptosis in wt NS, Rac1^fl/fl^ or Rac1 cKO retinas 24 h after rapamycin treatment (Rapa24h). The insets in the right of Rac1^fl/fl^ Rapa24h images are the enlarged images of the white squares. Scale bar: 50 µm for all images except 20 µm in the enlarged images. **d** Bar chart showing the average number of apoptotic RGCs in whole-mounted retinas under the conditions as shown in **c**. Error bars represent S.E.M., and *n* = 5 for all the groups. **P < 0.01 vs. wt NS; ^#^*P* < 0.05 vs. Rac1^fl/fl^ Rapa24h.

To further demonstrate the modulatory roles of Rac1 on RGC autophagy and apoptosis, we injected the mTOR inhibitor rapamycin intravitreally into mice. Rapamycin significantly increased the number of autophagosomes at 12 h and apoptotic RGCs at 24 h in Rac1^fl/fl^ retinas; these effects were blocked by Rac1 deletion (Fig. 7).

**Fig. 8:**
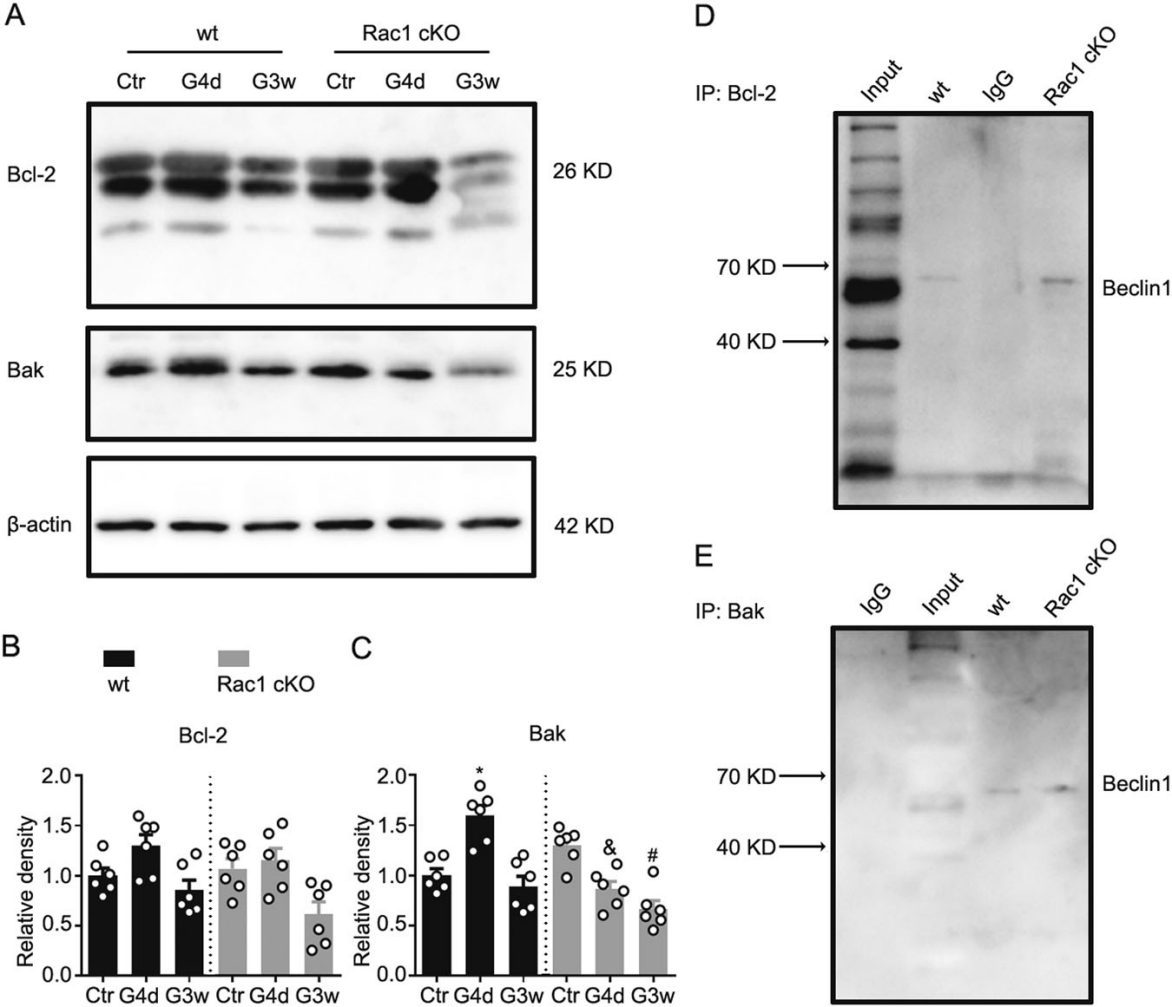
Rac1 regulates the expression of Bak in COH retinas. **a** Representative images showing the changes in Bcl-2 and Bak protein expression in wt and Rac1 cKO retinas with or without COH at G4d and G3w, respectively. **b**, **c** Bar charts summarizing the average densitometric quantification of immunoreactive bands of **b** Bcl-2 and **c** Bak under the conditions as shown in **a**. All data are normalized to their corresponding β-actin data and then to Ctr. Error bars represent S.E.M., and *n* = 6 for all groups. *P < 0.05, ^#^*P* < 0.001 vs. corresponding Ctr; ^&^*P* < 0.05 vs. G4d in wt group. **d**, **e** Co-IP experiments showing the interactions of Beclin1 with **d** Bcl-2 or **e** Bak in wt and Rac1 cKO retinas. Note that Rac1 cKO did not interfere with the cross talk of autophagy (Beclin1) and apoptosis (Bak).

Then we explored how Rac1 regulates autophagy and apoptosis in the COH retina. Anti-apoptotic factor Bcl-2 expression showed no significant changes in COH retinas of either wt or Rac1 cKO mice (Fig. 8a, b). By contrast, pro-apoptotic factor Bak expression increased significantly at G4d and returned to the control level at G3w in wt mice. Rac1 deletion inhibited Bak expression in COH mice (Fig. 8a, c). Furthermore, co-IP assays showed that both Bcl-2 and Bak interacted directly with Beclin1; Rac1 deletion did not affect these interactions (Fig. 8d, e). These results suggest that Rac1 modulates autophagy and apoptosis by regulating mTOR and Bak but not Bcl-2.

## Discussion

In the present work, a major finding is that biphasic autophagy in experimental glaucomatous RGCs functioned dynamically during cell injury: promoting RGC apoptosis in the early stages (G4d) and resulting in RGC death in the second stage (G3w). Activated Rac1 exacerbated RGC injury in COH retinas, as evidenced by Rac1 deletion in RGCs significantly attenuating RGC apoptosis through blocking autophagy, which occurred primarily via regulation of mTOR activity and Bak.

### The relationship between autophagy and apoptosis in glaucomatous RGCs

What is the role of autophagy in glaucomatous cell apoptosis? Autophagy and apoptosis dynamically occur in the glaucomatous pathological process, and one of them dominates the other, in a sequence that autophagy precedes apoptosis, as evidenced by our results of COH and rapamycin-treated mice, which is consistent with reports in other cells^29–32^. Autophagy may also have different roles in RGC apoptosis during glaucoma^33^. In this study, we observed that biphasic autophagy in COH retinas was activated, manifested by the elevation of LC3-II/I and Beclin1, and a further increase of protein levels after treatment with Baf-A1 (a blocker of lysosomal degradation). Considering the result of Baf-A1 treatment and the number of autolysosomes gradually increasing during IOP elevation (Figs. 1 and 2), the increased level of p62, which is well known as autophagy-specific substrate^34^, in COH retinas was due to the activation of autophagy, but not the blockage of autophagic flux. The first autophagic flux (G4d) may be an endogenous protective mechanism to promote the repair of damaged organelles, increasing the probability of cell survival; RGCs that fail to undergo successful repair might die through the apoptotic pathway. Autophagic cells in the second autophagic flux (G3w) underwent death directly without undergoing the apoptotic process. The nuclei, where autophagosomes are located, became gradually “shadowed” at G3w, which suggests that autophagic RGCs tend to die (Fig. 1). Autophagy overactivation, and the consequent self-digestion, is associated with cellular death^35^. Autophagy might control an apoptosis “threshold” and its alternating effects are based on the cross talk with apoptotic signaling molecules, such as Beclin1 with Bax or Bcl-2, and Atgs with caspases^36–39^. Although the final fate of cells is death, the timing of cell death may vary. It seems that the cells that die via apoptosis may undergo milder damage than those that die directly via autophagy. Cells with mild damage can function for a longer period of time because apoptotic cells can still perform their physiological functions until phosphatidylserine (PS) residues are exposed. Exposure of PS emits “eat me” signals to neighboring cells^40^, and the clearance of apoptotic cells occurs via phagocytosis and lysosomes^41–43^. Some reports have indicated that the accumulation of autophagic vacuoles alone is not lethal and that these cells can still recover upon withdrawal of the death-inducing stimulus^43,44^. Inhibiting the first peak of autophagy at G4d resulted in a reduction in RGC apoptosis; the opposite effect was observed after the second peak of autophagy was blocked at G3w (Fig. 5), which suggests that the second autophagic flux mainly has a clearance function. Therefore, regulating the first autophagic flux is more important for RGC survival.

### The role of Rac1-mediated signaling in RGC autophagy of COH mice

The molecular mechanism of autophagy is complex and highly conserved. Autophagy requires intracellular molecular trafficking and signaling pathways that regulate the cytoskeleton and differentiation^17,45,46^. Rac1 is an important molecule for remodeling the cytoskeleton and has an important role in cell survival^47^. Activation of Rac1 inhibits autophagy by competing with LC3 to bind on the neighboring domain in Armus, which regulates autolysosome biogenesis^17^. However, in this study, we found that Rac1 cKO accelerated autophagosome-lysosome fusion at G4d (Fig. 2) and reduced the number of autophagosomes in COH retinas by inhibiting autophagy-related protein expression (Fig. 3). Rac1 serves as a central hub in integrating different signaling pathways, and exert different functions due to targeting different downstream molecules^48^.

mTOR is a key central regulator in autophagy induction^1^. Rapamycin, a specific inhibitor of mTOR, converts the autophagy-specific protein phosphorylase pattern, contributes to the dephosphorylation of Atg13, and activation of other autophagy-related genes, and promotes autophagy^49^. In this study, we found that mTOR was downregulated in COH retinas, which is consistent with the upregulation of autophagy-related proteins and active Rac1 in the time course (Fig. 1). In addition, Rac1 can directly bind mTOR or indirectly regulate mTOR through its guanylate exchange factor P-Rex1^14,15^. In a model of rapamycin-induced autophagy, without other input factors interfering, Rac1 cKO significantly reduced the number of apoptotic RGCs (Fig. 7), which suggests that Rac1 deletion inhibits autophagy, thus contributing to neuroprotection. The effects of Rac1 deletion on RGC apoptosis and autophagy are mediated by inhibiting the expression of Beclin1, LC3, and Bak. Beclin1 is an autophagy effector that was originally identified as a Bcl-2-interacting protein. The pro-apoptotic factor Bak and anti-apoptotic factor Bcl-2 are both BH3-only members^50–52^, and the relative activities of BH3 proteins and initiator and/or executioner caspases decides that Beclin1 could either promote or inhibit autophagy^39^. Our co-IP results demonstrated an interaction between Beclin1 and Bak or Bcl-2 in both wt and Rac1 cKO mice. It suggests that Rac1 deletion does not interrupt the cross talk of beclin1 and BH3-only members.

In summary, Rac1 deletion in RGCs inhibits apoptosis in COH retinas by accelerating autolysosome formation, eliminating p62, as well as inhibiting Bak.

## Supplementary information

Supplementary Table 1
