# Supplementary material for: Rac1 conditional deletion attenuates retinal ganglion cell apoptosis by accelerating autophagic flux in a mouse model of chronic ocular hypertension
Source: Cell Death Dis. 2020 Sep 10;11(9):734. doi: 10.1038/s41419-020-02951-7 (PMC7484783; doi:10.1038/s41419-020-02951-7)
Supplement: Supplementary file 1 — Supplementary Table 1 [file 41419_2020_2951_MOESM1_ESM.docx]

**Supplementary Information for**

Rac1 conditional deletion attenuates retinal ganglion cell apoptosis by accelerating autophagic flux in a mouse model of chronic ocular hypertension

Meng-Lu Zhang*, Guo-Li Zhao*, Yu Hou, Shu-Min Zhong, Lin-Jie Xu, Fang Li, Wei-Ran Niu, Fei Yuan, Xiong-Li Yang, Zhongfeng Wang#, Yanying Miao#.

Department of Ophthalmology, State Key Laboratory of Medical Neurobiology and MOE Frontiers Center for Brain Science, Institutes of Brain Science, Zhongshan Hospital, Fudan University, Shanghai 200032, China

*These authors contributed equally to this work.

#Correspondence: Y. Miao and Z. Wang. Emails: yymiao@fudan.edu.cn (Y.M.) or zfwang@fudan.edu.cn (Z.W.).

**Supplementary Information**

All the antibodies used in the experiments of Western blotting and double immunofluorescent staining were summarized in Supplementary Table S1.

**Supplementary Table S1** Antibodies used in the experiments.

|  | **Company** | **Cat.#** | **Dilution** |
| --- | --- | --- | --- |
| **Western blotting**  β-actin | Sigma-Aldrich (MO, USA) | A5441 | 1:10000 |
| Beclin1 | Sigma-Aldrich or  Invitrogen/Thermo Fisher Scientific ( Waltham, MA, USA) | PRS3613 or  PA1-16857 | 1:1000  1:1000 |
| Rac1 | Abcam (Cambridge, MA, USA) | ab187108; ab33186 | 1:500 |
| active Rac1 | NewEast Biosciences (PA, USA) | 26903 | 1:500 |
| Bcl-2 | Invitrogen/Thermo Fisher Scientific | MA5-11757 | 1:50 |
| LC3 | Cell Signaling Technology (Danvers, MA, USA) | 12741 | 1:1000  1:1000 |
| p62 | Cell Signaling Technology | 5114 | 1:1000 |
| mTOR | Cell Signaling Technology | 2983 | 1:1000 |
| phospho-mTOR | Cell Signaling Technology | 2971 | 1:1000 |
| Bak | Cell Signaling Technology | 12105 | 1:1000 |
| HRP-conjugated IgG | Jackson ImmunoResearch Laboratories (Inc. West-Groove, PA, USA) | 711-035-152; 715-035-150 | 1:8000 |
| **Double immunofluorescent staining**  LAMP1 | Abcam | ab208943 | 1:100 |
| anti-LC3B | Cell Signaling Technology | 83506 | 1:400 |
| Cy3-conjugated IgG | Jackson ImmunoResearch Labs | 715-165-152 | 1:400 |
| Alexa Fluor 488 -conjugated IgG | Jackson ImmunoResearch Labs | 711-545-150 | 1:400 |
